# Cutaneous metastasis emerging during chemotherapy and progressing during immunotherapy for urothelial carcinoma

**DOI:** 10.1002/iju5.12350

**Published:** 2021-07-18

**Authors:** Isamu Otsuka, Takashi Ueno, Naoki Terada, Shoichiro Mukai, Tsuyoshi Fukushima, Takumi Kiwaki, Masafumi Nagano

**Affiliations:** ^1^ Department of Urology Fujimoto General Hospital Miyakonojo Departments of Japan; ^2^ Departments of Urology Faculty of Medicine University of Miyazaki Kiyotake Miyazaki Japan; ^3^ Department of Pathology Faculty of Medicine University of Miyazaki Kiyotake Miyazaki Japan

**Keywords:** chemotherapy, cutaneous metastasis, immune checkpoint inhibitor, urothelial carcinoma

## Abstract

**Introduction:**

Cutaneous metastasis of urothelial carcinoma is uncommon. We experienced a case of cutaneous metastasis that emerged during chemotherapy and progressed rapidly during immunotherapy for bladder tumor with lymph node metastasis.

**Case presentation:**

A 77‐year‐old female patient received chemotherapy using gemcitabine and cisplatin for bladder urothelial carcinoma with para‐aortic and pelvic lymph node metastases (clinical stage T2N1M1). After eight courses of chemotherapy, skin rash appeared in the lower abdomen with leg edema caused by multiple lymphadenopathy. Skin biopsy revealed cutaneous metastasis of urothelial carcinoma. The cutaneous tumor progressed rapidly and the patient died 5 weeks after the start of second‐line pembrolizumab treatment.

**Conclusion:**

A patient with cutaneous metastasis of urothelial carcinoma that emerged during chemotherapy had poor prognosis and lack of efficacy of an immune checkpoint inhibitor.

**Abbreviations**
CK: cytokeratin
CT: computed tomography

**Keynote message**

We report a rare case of cutaneous metastasis of urothelial carcinoma that progressed rapidly under treatment with immune checkpoint inhibitor. Pathological examination should be considered in cases suspicious for cutaneous metastasis.

## Introduction

Lymph nodes, bone, lungs, liver, and peritoneum are common metastatic sites of bladder urothelial carcinoma.^1^ Cutaneous metastasis of urothelial carcinoma is uncommon (0.84%).^2^ It is reported that the median survival time of patients with cutaneous metastasis is <6 months.^3^ We report a case of cutaneous metastasis of urothelial carcinoma that emerged during chemotherapy and progressed rapidly during immunotherapy for bladder tumor with lymph node metastasis.

## Case presentation

**Fig. 1:**
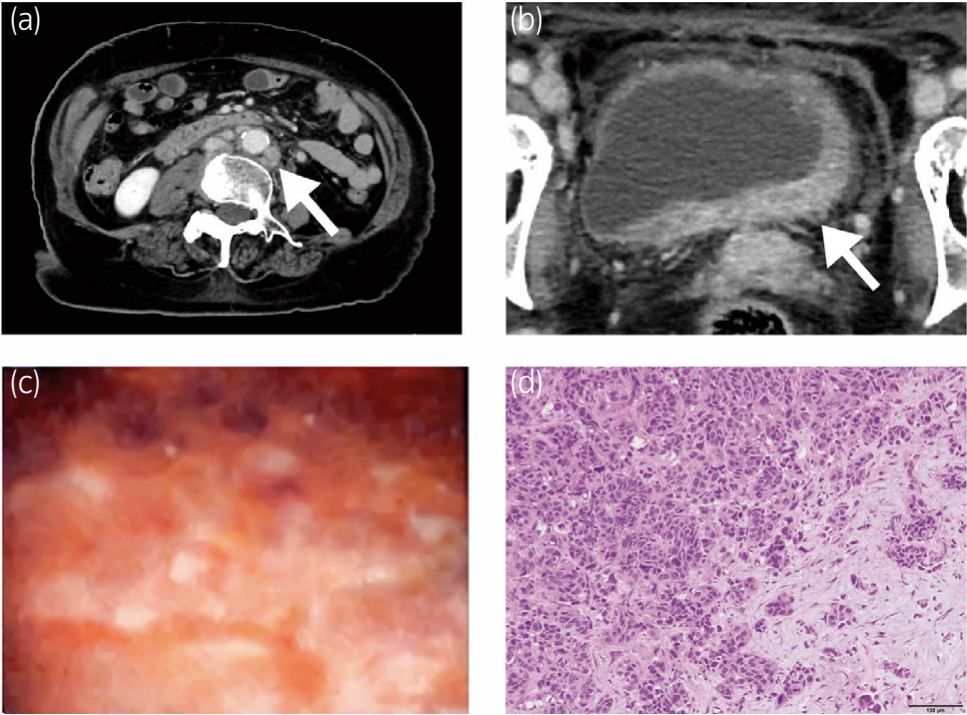
(a) Para‐aortic lymph node swelling in upper abdominal computed tomography (CT) (arrow). (b) Thickening of the bladder wall enhanced by contrast medium in lower abdominal CT (arrow). (c) Mucosal swelling and redness revealed by cystoscopy examination. (d) High‐grade invasive urothelial carcinoma with tumor cells infiltrating the stroma in a nested fashion (hematoxylin and eosin staining; scale bar 100 μm).

**Fig. 2:**
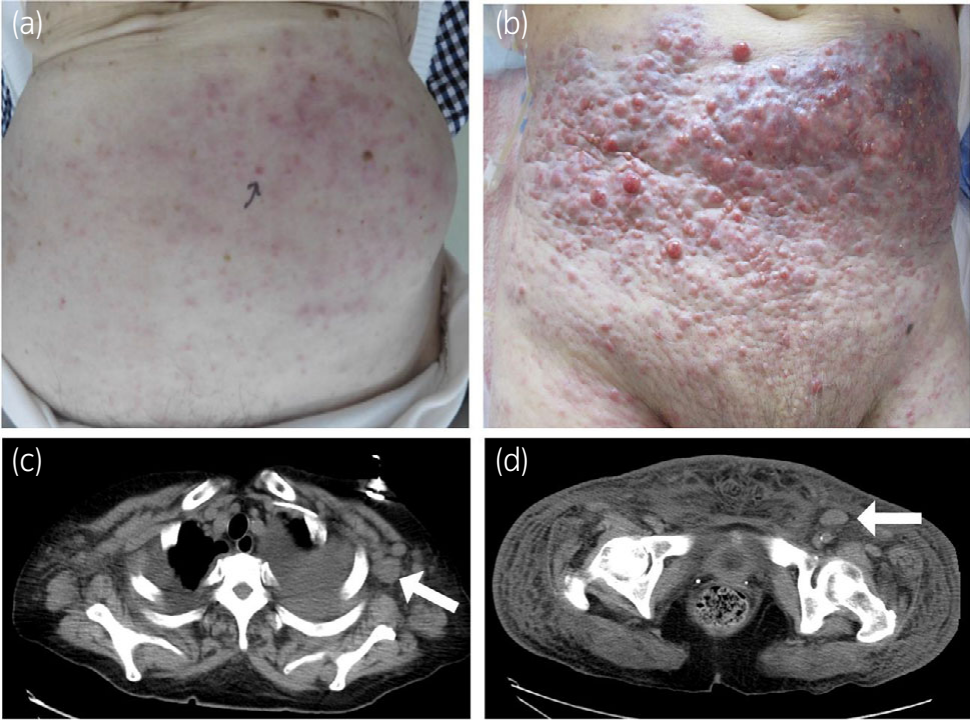
(a) Skin lesions on the lower abdominal wall before pembrolizumab therapy harboring multiple nodules with redness. (b) Skin lesions on the lower abdominal wall after pembrolizumab therapy. Infiltrative erythema and nodules with fusion. (c) Left axillary lymph node swelling on the computed tomography (CT) before pembrolizumab therapy (arrow). (d) Left inguinal lymph node swelling and subcutaneous infiltration on CT before pembrolizumab therapy (arrow).

**Fig. 3:**
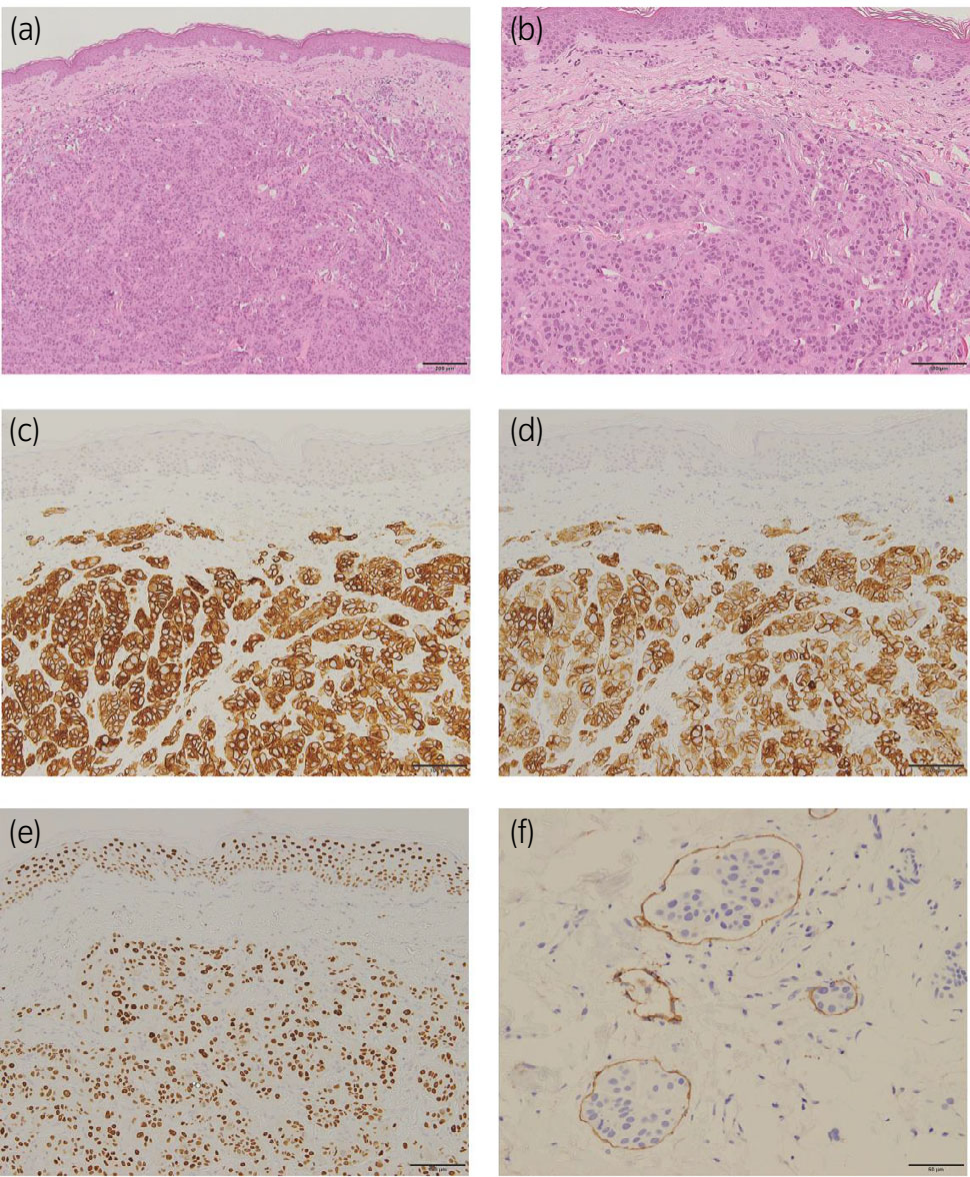
Histopathologic findings of skin biopsy. (a) Tumor cells proliferating in subcutaneous stromal tissue in a nested pattern hematoxylin and eosin staining; scale bar 200 μm). (b) Tumor cells resembling bladder tumor (scale bar 100 μm). (c) Immunostaining of cytokeratin (CK7) (scale bar 100 μm). (d) Immunostaining of CK20 (scale bar 100 μm). (e) Immunostaining of GATA3 (scale bar 100 μm). (f) Lymphatic infiltration with D2‐40 immunostaining (scale bar 50 μm).

A 77‐year‐old woman presented with pollakiuria. Ultrasonography showed left hydronephrosis. Computed tomography (CT) showed thickening of the bladder wall with para‐aortic and bilateral external iliac lymphadenopathy (Fig. 1a,b). Under cystoscopic examination, only abnormal mucosa was identified around the bladder neck without any tumor (Fig. 1c). Histopathological examination by transurethral biopsy revealed high‐grade invasive urothelial carcinoma (Fig. 1d). The patient was diagnosed with bladder tumor of clinical stage T2N1M1. Chemotherapy with cisplatin (56 mg/m^2^, day 2) and gemcitabine (1000 mg/m^2^, days 1 and 8) was administered. The dose of cisplatin was reduced by 20% because of old age. Lymphadenopathy did not change during treatment. After eight courses of chemotherapy, bilateral lower leg edema and skin rash in the lower abdomen appeared (Fig. 2a). Skin lesion biopsy revealed proliferation of tumor cells with lymphatic infiltration. The tumor cells resembled those in the previous specimen of the bladder tumor with positive expression of cytokeratin (CK)7, CK20 and GATA3 (Fig. 3). Thus, we diagnosed this lesion as cutaneous metastasis of urothelial carcinoma. CT revealed progression of bilateral axillary and inguinal lymphadenopathy (Fig. 2b,c). Pembrolizumab (200 mg) was administered as second‐line therapy soon after diagnosis of cutaneous metastasis. Two weeks after initiation of pembrolizumab, the patient was admitted with severe whole‐body edema and abdominal pain. The cutaneous tumor progressed rapidly (Fig. 2d) and she presented with anasarca. CT showed pleural effusion, probably caused by lung metastasis, without any interstitial pneumonia. Serum biomarkers for cytokine storms were within the normal range (interleukin‐6: 167 U/mL, pulmonary surfactant protein D: 15.7 ng/mL). This indicated that the symptom was not an adverse event of pembrolizumab therapy but was caused by tumor progression. She went into respiratory failure and died 5 weeks after initiation of pembrolizumab treatment.

## Discussion

Skin is a rare site of metastasis from any type of cancer.^1^ It is generally considered that the immune mechanisms of the skin prevent cutaneous metastasis of cancer cells.^2^ The incidence of cutaneous metastases from all malignancies is 0.2%–10.4%, and the top two primary cancers are breast and lung.^4^ The rate of cutaneous metastasis of bladder tumor is reported to be 0.84%.^2^ In elderly patients, the rate of cutaneous metastasis tends to increase, probably because of deterioration of the immune system.^5^ Bladder tumor commonly occurrs in elderly patients. With the advent of new treatments for urothelial cancer, the lifespan of cancer‐bearing patients will potentially be extended. Along with this trend, the number of patients with skin metastasis may increase in future.

The clinical appearance of cutaneous metastases is categorized into nodular or inflammatory type.^3^ The present case might be categorized into inflammatory type. The mechanisms for cutaneous metastases are direct invasion, lymphatic spread, or hematogenous spread.^3^ Cutaneous metastasis of bladder tumor commonly occurrs in the lower abdomen, as in the present case. It might be caused by lymphatic spread with altered lymphatic drainage routes from the urinary bladder by multiple lymphadenopathy. Our patient showed atypical metastases to inguinal lymph nodes. Cancer cells might have migrated from the inguinal lesions to the lower abdominal skin.

Our patient had only a small local bladder tumor with lymph node metastasis at the start of chemotherapy. Infiltrating urothelial carcinoma such as this is generally considered to have poor prognosis without any visceral metastasis. The association of infiltrating urothelial carcinoma with the occurrence of cutaneous metastasis has not been reported previously. The initial appearance of the skin lesion looked like a rash caused by chemotherapeutic reagents. Skin biopsy was performed because the patient had progressive lymphadenopathy with foot edema. In the era of immune checkpoint inhibitors, Stevens‐Johnson syndrome, erythema multiforme, and pemphigoid can also occur as rare adverse events, and their differential diagnosis is also necessary. Our case indicated that pathological diagnosis should be considered in cases suspicious for cutaneous metastasis. For the pathological diagnosis of cutaneous metastasis, we performed skin biopsy. Dermoscopy is an essential tool for attempting early diagnosis of melanoma and has also been used for other skin lesions.^6^ Thus, dermoscopy can be examined for skin lesion more noninvasively, which results in diagnosis by skin biopsy when cutaneous metastasis is suspected.

It is reported that most patients with cutaneous metastasis of bladder tumor die within 6 months after diagnosis.^3^ Management strategies have not been clearly defined for cutaneous metastasis of urothelial carcinoma because of the small number of patients and their poor prognosis.^7^ It is reported that metastasectomy is effective for cutaneous metastasis,^8^ although only for local metastasis.^9^ Our case had diffuse skin lesions that made it difficult to perform metastasectomy. It is reported that standard platinum‐based chemotherapy is not effective for cutaneous metastasis of urothelial carcinoma.^2^ Uemura *et al*. reported a case of hematogenous spread of cutaneous metastasis for which chemotherapy with gemcitabine plus paclitaxel was effective.^10^ Recently, immune checkpoint inhibitors have been widely incorporated into the treatment of advanced urothelial carcinoma refractory to chemotherapy.^11^ Ohashi *et al*. reported that cutaneous metastasis of lung cancer shrank markedly after fifth‐line treatment with nivolumab.^12^ However, Hasan *et al*. reported no efficacy for cisplatin and gemcitabine followed by atezolizumab for cutaneous metastasis of bladder urothelial carcinoma.^13^ In our case, cutaneous metastasis emerged during treatment with platinum‐based chemotherapy, and progressed rapidly during immunotherapy with pembrolizumab. All these findings suggest that immune checkpoint inhibitors might not be an effective treatment option for cutaneous metastasis of urothelial carcinoma. Further accumulation of case reports is needed to evaluate the efficacy of immunotherapy for skin metastasis of urothelial carcinoma.

## Conclusion

We present a case in which cutaneous metastasis emerged during chemotherapy and progressed rapidly during immunotherapy for invasive bladder tumor with lymph node metastasis. Cutaneous metastasis of urothelial carcinoma is aggressive and immune checkpoint inhibitors might not be effective. Pathological examination should be considered in cases suspicious for cutaneous metastasis to decide the treatment strategy for urothelial carcinoma.

## Conflict of interest

The authors declare no conflict of interest.

## Approval of the research protocol by an Institutional Reviewer Board

Not applicable.

## Informed consent

Informed consent was obtained from the patient for publication of this case report and accompanying images.

## Registry and the Registration No. of the study/trial

Not applicable.
